# Structural and haemostatic features of pharmaceutical heparins from different animal sources: challenges to define thresholds separating distinct drugs

**DOI:** 10.1038/srep35619

**Published:** 2016-10-18

**Authors:** Ana M. F. Tovar, Gustavo R. C. Santos, Nina V. Capillé, Adriana A. Piquet, Bianca F. Glauser, Mariana S. Pereira, Eduardo Vilanova, Paulo A. S. Mourão

**Affiliations:** 1 Laboratório de Tecido Conjuntivo, Hospital Universitário Clementino Fraga Filho and Instituto de Bioquímica Médica Leopoldo de Meis, Universidade Federal do Rio de Janeiro (UFRJ), Rio de Janeiro 21941-913, Brazil

## Abstract

Heparins extracted from different animal sources have been conventionally considered effective anticoagulant and antithrombotic agents despite of their pharmacological dissimilarities. We performed herein a systematic analysis on the physicochemical properties, disaccharide composition, *in vitro* anticoagulant potency and *in vivo* antithrombotic and bleeding effects of several batches of pharmaceutical grade heparins obtained from porcine intestine, bovine intestine and bovine lung. Each of these three heparin types unambiguously presented differences in their chemical structures, physicochemical properties and/or haemostatic effects. We also prepared derivatives of these heparins with similar molecular weight differing exclusively in their disaccharide composition. The derivatives from porcine intestinal and bovine lung heparins were structurally more similar with each other and hence presented close anticoagulant activities whereas the derivative from bovine intestinal heparin had a higher proportion of 6-desulfated α-glucosamine units and about half anticoagulant activity. Our findings reasonably indicate that pharmaceutical preparations of heparin from different animal sources constitute distinct drugs, thus requiring specific regulatory rules and therapeutic evaluations.

Heparin is widely employed in medicine as anticoagulant and antithrombotic agent. The clinical use of heparin has started about 65 years ago, which places it within the oldest biologic drugs still in use^1^; furthermore, it is the second leading biologics currently in use, just behind insulin^2^. Heparin is a unique sulfated glycosaminoglycan (GAG), with one of the highest anionic charges of natural biomolecules, and can be considered as a major representative of a carbohydrate-based drug^3^. Its use is essential for procedures of extracorporeal circulation during cardiovascular surgeries and hemodialysis^4^. The mechanism of action of heparin on the coagulation system is well characterized at molecular level and acts by carbohydrate-protein interaction resulting in proteases inhibition mediated by serpins^1^.

World consumption of heparin has been increasing significantly mainly due to more precise diagnosis of thromboembolic diseases and the increasing importance given to their prevention. However, the heparin production is restricted by its dependence of animal tissues as basic raw material. Different tissues from different species of mammals were already employed over the years for manufacturing heparin with pharmaceutical purposes; originally from dog liver, then from bovine lung and lately from porcine intestine^5^. In some Latin American and Islamic countries bovine intestine is also a source of heparin^6^. Currently, Chinese industries produce approximately 60% of the heparin consumed in the world using porcine intestine as raw material^7^.

The current production of heparin mostly based on a single animal source and concentrated in a single country raises a “supply insecurity”, which lead the FDA – USA to evaluate the reintroduction of lung or intestine bovine heparins^8^. Possibly, such argument will be extended to other countries, demanding new information and regulatory rules on the chemical structures and haemostatic features of heparins obtained from sources other than porcine intestine.

Heparin is a linear polysaccharide mostly constituted for 4-linked units of α-L-iduronic acid 2-sulfate (α-IdA-2S) and α-D-glucosamine *N*,6-disulfated (α-GlcN-*N*,6diS). Other minority units found in the molecule are non-sulfated α-L-iduronic acid, α-D-glucosamine *N*-acetylated or *N*, 3, 6-trisulfated, and β-D-glucuronic acid residues (not epimerized to α-L-iduronic acid). The proportion of these units in the heparin polymer directly relates to the tissue used for its extraction, notably: the proportion of *N*-acetylated α-D-glucosamine residues is lower in bovine lung heparin^9^ and the amount of 6-sulfated α-D-glucosamine units is significantly lower in bovine than in porcine intestinal heparin^10^,^11^. In addition to these structural differences, heparins obtained from different sources also differ in their average molecular weight^9^.

The structural variations among heparins obtained from different sources induce significant changes on their anticoagulant effects, including the dosage required for the prevention of thrombosis and leading to a bleeding adverse effect. For example, it is necessary to use higher quantity (in weight basis) of bovine intestinal than porcine intestinal heparin to achieve the same anticoagulant and antithrombotic effects. These differences have direct implications in the clinical use of heparin, such as the higher dosages of protamine necessary to neutralize bovine than porcine intestinal heparins^10^,^11^.

The objective of the present study was to answer two essential questions involving the production and therapeutic use of heparin: (1) Are heparins obtained from different sources distinct drugs? (2) Which are the analytical thresholds separating these distinct heparins? To answer these questions, we undertook a systematic analysis on physicochemical properties, disaccharide composition*, in vitro* anticoagulant activity and *in vivo* antithrombotic and bleeding effects of pharmaceutical heparins obtained from porcine intestine (HP-I), bovine intestine (HB-I) and bovine lung (HB-L). We also prepared and analyzed derivatives of these heparins with similar molecular weight but differing in their disaccharide units. Our findings allowed us to clarify many aspects of these questions concerning the pharmacological features of heparins produced from different animal sources.

## Results

### Physicochemical Properties

**Figure 1:**
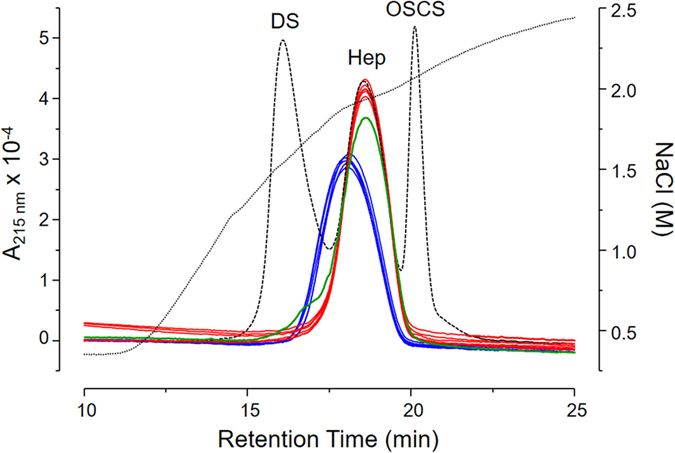
Anion-exchange chromatography of heparins from different animal sources. HP-I (red line), HB-I (blue line) and HP-L (green line) were eluted from an IonPac AS11-HC column through a linear gradient of 0.4 → 2.5 M NaCl (dotted line). Dashed line shows the elution profile of a standard mixture containing 50 μg dermatan sulfate (DS), 200 μg 6^th^ International Heparin Standard from NIBISC (Hep) and 30 μg oversulfated chondroitin sulfate (OSCS). The eluents were monitored via UV (A_215nm_).

Pharmaceutical heparins obtained from different animal sources were initially analyzed via anion exchange chromatography. HB-I (Fig. 1, blue lines) elutes as a broader peak and with lower concentration of NaCl than HP-I (Fig. 1, red lines) or HB-L (Fig. 1, green line). One hundred and seventeen batches of HB-I and 25 of HP-I were analyzed, showing reproducible elution profiles as those illustrated in Fig. 1. In contrast, only one batch of HB-L was analyzed; this is because its production was ended in the 1980’s and thus commercially unavailable. HB-I and HP-I batches showed no contaminations with dermatan sulfate or oversulfated chondroitin sulfate while a discrete contamination with dermatan sulfate (<5%) was observed in the HB-L batch (Fig. 1). We also quantitatively evaluated the capacity of retention of these heparins on the anion exchange column at different NaCl concentrations. With NaCl concentrations up to 1.9 M, 56.2% of HB-I (6 batches), 27.7% of HP-I (5 batches) and 35.5% of HB-L (1 batch) were eluted from the column (Supplementary Table S1). This clearly demonstrates that the different heparins have distinct ionic properties, especially those extracted from bovine intestine.

**Figure 2:**
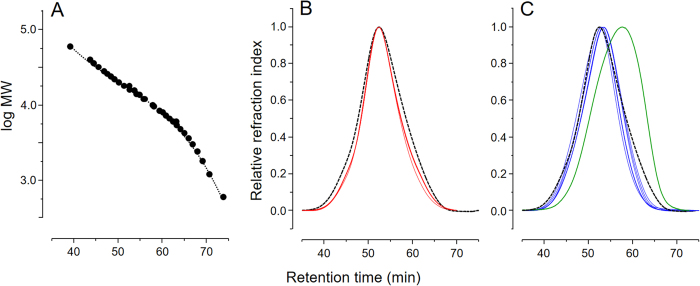
Gel permeation chromatography of heparins from different animal sources. Molecular weight distributions of calibrants (**A**), HP-I (red lines in panel B) and HB-I and HB-L (blue and green lines in panel C, respectively) were determined using a set of TSK gel G4000 SW/G3000 SW columns. Retention times and relative molecular weight of the calibrants (low-molecular-weight heparin - NIBSC and heparin sodium - USP) were used to derive the calibration curve (fit to a third order polynomial) depicted in the panel A. Dashed black lines on panels B,C show the elution of the heparin sodium molecular-weight calibrant from USP. The eluents were monitored via differential refractive index.

The molecular weight of these heparins was investigated via gel permeation chromatography, using a set of columns previously calibrated with the heparin sodium molecular-weight calibrant from USP and the 2nd low-molecular-weight heparin molecular-weight standard from NIBSC (Fig. 2A). HP-I and HB-I batches (Fig. 2B, red lines and Fig. 2C, blue lines, respectively) showed very low variability within and between themselves unlike HB-L, which had a noticeably reduced molecular weight distribution (Fig. 2C, green line). The *M*_*w*_, *M*_*p*_, *M*_*n*_ and PD of these heparins are list in Table 1. HP-I and HB-I are more alike one each other, nevertheless differing significantly (P < 0.05) in their *M*_*p*_, *M*_*n*_ and PD values (Table 1). All the six batches of HP-I comply with the requirements for molecular weight range defined by the USP (Supplementary Table S2). Only one within the six batches of HB-I analyzed did not comply with USP recommendations regarding the ratio *M* 8,000 → 16,000 Da/*M* 16,000 → 24,000 Da^12^.

### Disaccharide composition

**Figure 3:**
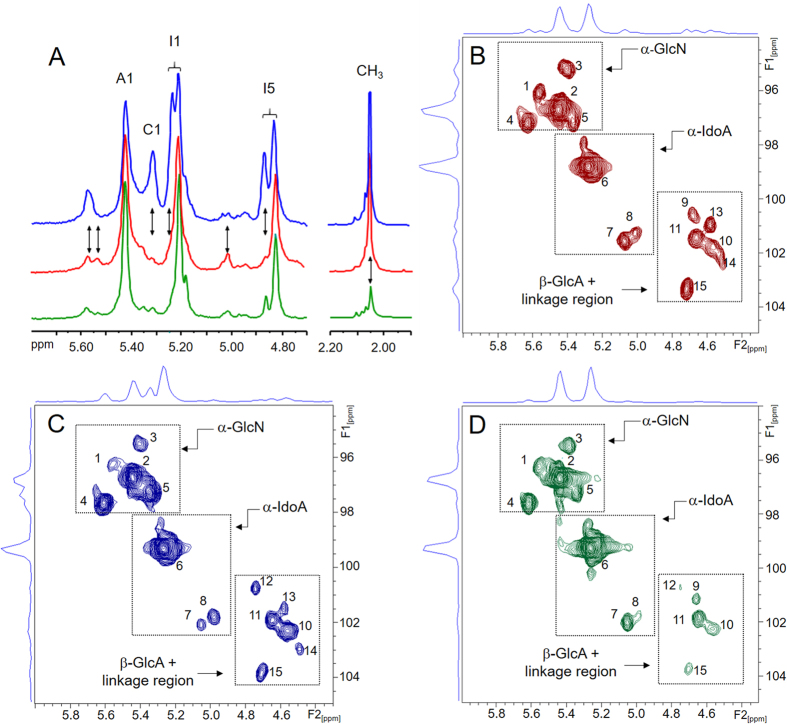
NMR spectra of heparins from different animal sources. 1D ^1^H spectra of the regions 4.70–5.70 and 1.90–2.20 ppm expansions (**A**) and strips of the anomeric region of 2D ^1^H - ^13^C HSQC NMR spectra (**B**–**D**) of HP-I (in red), HB-I (in blue) and HB-L (in green). The labels in the panel A indicate signals of the anomeric protons of α-glucosamine *N*,6 disulfated (A1) and α-glucosamine *N*-sulfated (C1); H1 (I1) and H5 (I5) of 2-sulfated α-iduronic acid; and CH_3_ to the *N*-acetyl group. The vertical arrows indicate the major differences among the spectra of the three types of heparin. The numbers in the panels B–D refer to the anomeric signals of the structures described in Table 2. The dotted squares in the Panels B–D enclose the regions of the spectra containing the anomeric signals of α-glucosamine (α-GlcN), α-iduronic acid (α-IdA) and β-glucuronic acid (β-GlcA) + units of the linkage region.

NMR analysis of the three heparins revealed some coincident signals on 1D ^1^H NMR spectra in the ranges of 4.8–5.6 and 1.9–2.2 ppm (Fig. 3A). Despite these analogies, HB-I showed significant structural differences in comparison with the other two heparin types (see vertical arrows in Fig. 3A); mainly with regard to its higher proportion of 6- desulfated α-glucosamine units, clearly identified by the anomeric signal at 5.31 ppm^10^,^11^. The lack of sulfate in the position 6 of α-glucosamine unit also results in a modest downfield shift of H1 and H5 of the neighbor 2-sulfated α-iduronic acid (Fig. 3A). In addition, HB-I contains a higher proportion of β-glucuronic acid linked to *N*-sulfated α-glucosamine (H1 at 5.58 ppm) and lower proportion of α-glucosamine *N*,3,6-trisulfated (H1 at 5.52 ppm) and non-sulfated α-iduronic acid (H1 at 5.01 ppm) (Fig. 3A). In contrast, HB-L and HP-I showed only subtle variations in their structures; the only noticeable difference was the low intensity of the *N*-acetyl signal at 2.06 ppm in HB-L, indicating lower proportion of *N*-acetylated α-glucosamine, as previously reported^9^.

**Figure 4:**
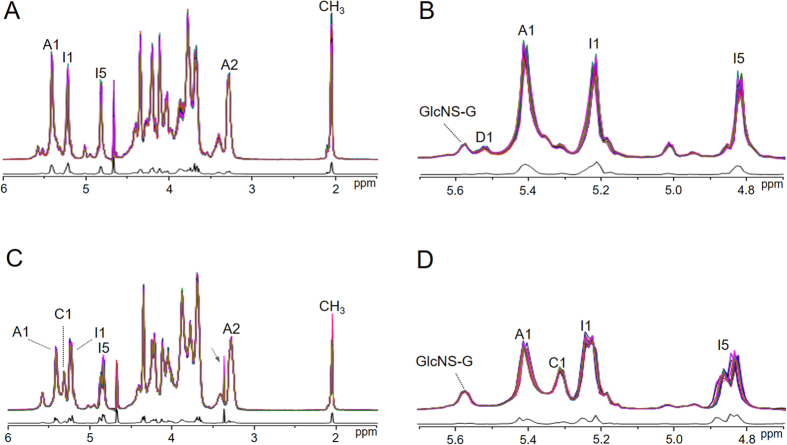
Weighted 1D ^1^H NMR. Spectra of 20 batches of each HP-I (**A**,**B**) and HB-I (**C**,**D**) were weighted and fitted using AMIX software to generate concatenate plots (colored lines) and their respective SD (black lines). Panels A–C and B–D show 6.0–1.0 and 5.7–4.7 ppm expansions, respectively. Labels in the panels indicate signals of the anomeric protons of α-glucosamine *N*,6 disulfated (A1), α-glucosamine *N*-sulfated (C1) and α-glucosamine *N*,3,6 trisulfated (D1), H2 of α-glucosamine (A2), H1 (I1) and H5 (I5) of 2-sulfated α-iduronic acid and CH_3_ to the *N*-acetyl group (CH_3_). GlcNS-G refers to the anomeric proton of α-glucosamine *N*-sulfate linked to β-glucuronic acid. The arrow in Panel C indicates a signal of residual solvent (methanol). The weighted spectra are coincident and the deviations regard to normal variations in the Lorentzian line widths of ^1^H NMR signals.

1D ^1^H NMR analysis performed with a large number of batches of HP-I and HB-I yielded 1D ^1^H spectra with high similarities within the batches and a marked difference between each heparin type. To further assess the regularity and reproducibility of the chemical composition of these heparins we weighted and fitted as concatenate plots the spectra of 20 batches of each HB-I and HP-I (Fig. 4). As expected, the average plots of HB-I and HP-I batches were fairly similar within and different between themselves (Fig. 4). Although these plots lack resolution to discriminate the double-peaks of H1 and H5 signals of the 2-sulfated α-iduronic acid units, they can be considered a useful analytical tool to compare multiple batches of heparin. Other relevant information derived from 1D ^1^H spectra was the level of purity of these heparins; we were able to detect mixtures of HB-I and HP-I up to 4% on weight basis (Supplementary Figure S1).

We further analyzed the different heparins via 2D NMR techniques. ^1^H - ^13^C HSQC spectra (Fig. 3B,D) were used to identify and quantify the constituent units of the heparins, including their minority components, as previously described^13^. These spectra are particularly useful to identify residues of β-D-glucuronic acid, which are superimposed by the HOD signal in 1D ^1^H spectra. Three distinct regions on the HSQC spectra were clearly ascribed to the anomeric signals of α-glucosamine, α-iduronic acid and β-glucuronic acid + units of the linkage region (Fig. 3B,D, dotted squares). The ^1^H and ^13^C chemical shifts of the anomeric units from each heparin type are available in the Supplementary Table S3. The constituent units of these heparins were qualitatively similar but differ in their proportions (Table 2), especially the units from HB-I, which showed: greater heterogeneity of α-glucosamine units, mainly because its 6-desulfation (signal 5, Table 2); higher proportion of *N*-sulfated α-glucosamine linked to β-glucuronic instead α-iduronic acid (signal 4); and lower content of β-glucuronic acid linked to *N*-glucosamine *N*,3,6-trisulfated (signal 9), a component of the binding region to antithrombin^14^. We also observed that HB-L has more uniform composition than HP-I and HB-I, especially due to the sulfation pattern of α-glucosamine units and lower content of β-glucuronic acid (Table 2).

The connection among the different α-glucosamine units and 2-sulfated α-iduronic acid of each heparin type was further investigated via phase-sensitive ^1^H-^1^H TOCSY (Supplementary Figure S2), which discern signals of intra-residue spin systems from those of inter-residue ROES^15^,^16^,^17^. This approach allows determining the saccharide sequence and linkage position through the correlation of anti-phase (indicated in red signals and their connections by the horizontal dotted lines) and in-phase (shown in blue signals and their connections indicated by the vertical dotted lines) (Supplementary Figure S2). Briefly, the most intense anti-phase signal shows the linkage in the subsequent sugar residue. The connection of the anomeric signal of 6-desulfated α-glucosamine (C1) and the two slightly down-shifted H1 and H5 signals of 2-sulfated α-iduronic acid (namely I1-C and I5-C, respectively) was previously speculated correlating their intensities^10^. Now we unambiguously demonstrated that C1 signal of 6-desulfated α-glucosamine and A1 signal of *N*,6-disulfated α-glucosamine connect with signals I1-C/I5-C and I1-A/I5-A, respectively, of 2-sulfated α-iduronic acid (Supplementary Figure S2). The set of 1D and 2D NMR analysis presented above clearly demonstrate that the disaccharide composition of HB-I markedly differs from those of HB-L and HP-I.

### Anticoagulant and antithrombotic activities and bleeding tendency

**Figure 5:**
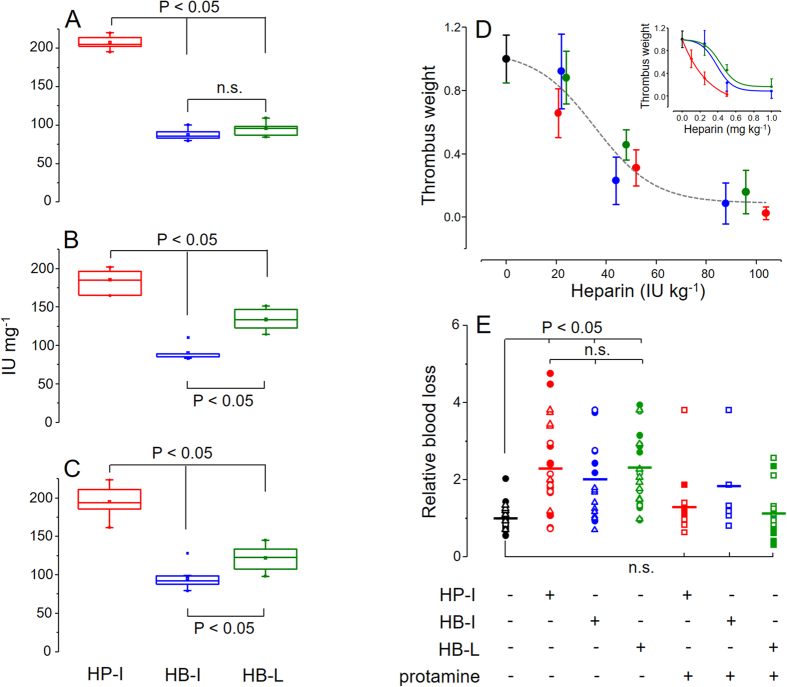
*In vitro a*nticoagulant and *in vivo* antithrombotic and bleeding effects of heparins from different animal sources. HP-I (in red), HB-I (in blue) and HB-L (in green) were evaluated *in vitro* on APTT (**A**), anti-FIIa (**B**) and anti-FXa (**C**) and *in vivo* on venous thrombosis (**D**) and bleeding tendency (**E**) assays. Data from the *in vitro* assays (panels A–C) were expressed as median (CI 95%) and mean (□) and compared via One-way ANOVA with Bonferroni post hoc test. The anticoagulant activities (IU mg^−1^, as dry weight) were determined based on standard curves obtained with the 6^th^ International Heparin Standard (NBISC). *In vivo* antithrombotic activities (normalized results) were expressed as IU kg^−1^ body weight (a single sigmoidal curve for the three heparin types) in panel D and as mg kg^−1^ body weight in panel D inset (mean ± SD, n ≥ 5). Bleeding tendencies (panel E) were expressed as ratios of blood loss of animals treated with the heparins (200 IU kg^−1^ body weight) and saline (control, in black). In part of the assays heparins were neutralized with 2 mg kg^−1^ body weight of protamine before the bleeding measurement. Full and empty squares, circles and triangles represent different experimentalists.

The *in vitro* anticoagulant potencies of the different heparins were determined based on clotting assays (APTT) (Fig. 5A) and on their anti-FIIa (Fig. 5B) and anti-FXa (Fig. 5C) activities. When analyzed separately, each heparin type presented similar (P > 0.05) specific activity (IU mg^−1^) on the three anticoagulant assays (Supplementary Table S4), except for the HB-L APTT, which was significantly lower than its anti-FIIa and anti-FXa activities (Supplementary Table S4). The comparison among the heparins through these assays revealed that HP-I has significantly higher anticoagulant potency (P < 0.05) than HB-I and HB-L, which also shows slightly but significant differences (P < 0.05) between them on anti-FIIa and anti-FXa assays (Fig. 5). This result indicates that despite of the structural resemblance between HP-I and HB-L, their *in vitro* anticoagulant activities are quite different.

To assess the *in vivo* antithrombotic activities of the three heparins we employed a classical venous model of experimental thrombosis after intravascular administration of the drug of interest^18^. These assays were performed using a single representative batch of each heparin. Higher doses of HB-I and HB-L than HP-I administered on weight basis (mg kg^−1^ body weight) were necessary to achieve a satisfactory antithrombotic effect (Fig. 5D, inset). However, the three heparin types generate similar dose *vs.* response curves with doses based on their anticoagulant activities (IU kg^−1^ body weight) (Fig. 5D). As expected, these *in vivo* assessments have reasonably reproduced the results obtained in the *in vitro* anticoagulant assays.

The bleeding tendencies of each heparin type were evaluated in rats^19^, before and after their neutralizations with protamine (Fig. 5E). Doses with similar anticoagulant potency (~200 IU kg^−1^ body weight) of one representative batch of each heparin type and saline (control) were intravascular administered and afterward the bleeding was measured. The hemorrhagic tendencies of the three heparins were similar among themselves (P > 0.05) and significant higher (P < 0.05) than the control (Fig. 5E). Animals treated with protamine (2 mg kg^−1^ body weight) showed no significant differences (P > 0.05) in comparison with the control. Therefore, the set of results presented above demonstrate that HP-I have higher anticoagulant and antithrombotic potencies than HB-I and HB-L (on weight basis) and similar bleeding tendencies with doses adjusted in IU.

### Preparation and analysis of heparins with similar molecular weight

**Figure 6:**
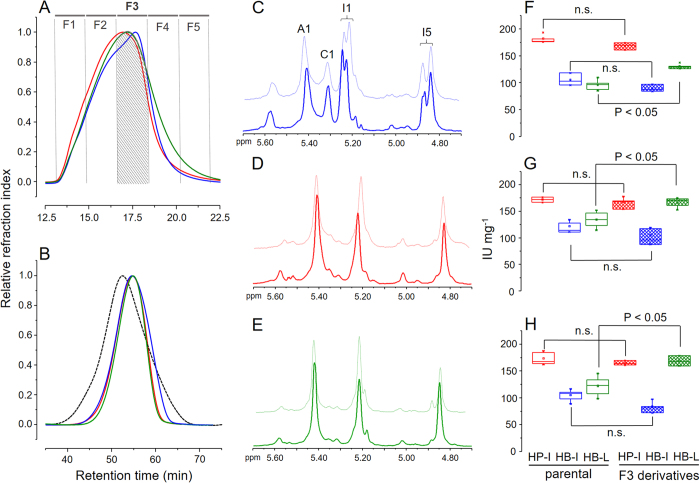
F3 derivatives preparation and analyses. (**A**) F3 derivatives from HP-I (in red), HB-I (in blue) and HB-P (in green) were prepared using a Superdex Peptide HR 10/30 column. The fraction F3 (crosshatched) yielded derivatives with similar molecular weight range. (**B**) Analytical assessment of F3 derivatives and heparin sodium USP references standard using a set of TSK gel G4000 SW/G3000 SW columns; for further details, see the legend for Fig. 2. 1D ^1^H NMR spectra of HP-I (in red), HB-I (in blue) and HB-P (in green) F3 derivatives (solid lines) and their unfractionated parental batches (dotted lines); for details about the signals see the legend for Fig. 3A. Anticoagulant activities of F3 derivatives (checked boxes) and their unfractionated parental batches (empty boxes) measured via APTT (**F**), anti-FIIa (**G**) and anti-FXa (**H**) assays. See legend for Fig. 5 for further information about the assays.

HP-I and HB-L share similarities on their disaccharide composition but differ on their anticoagulant potencies. The most notable difference between these two heparins is their molecular weight (Fig. 2, Table 1); such difference can directly influence the anticoagulant effects of heparin^20^. To confirm if the anticoagulant potency of HB-L is impaired by its molecular weight we prepared derivatives from the three heparin types with similar molecular weight range using a semi-preparative gel permeation column (Fig. 6A). One batch of each heparin type was pooled into five distinct fractions (F1 to F5), of which the fraction F3 yielded derivatives with similar molecular weight range and closest *M*_*w*_, *M*_*p*_, *M*_*n*_ and PD values (Fig. 6A, Table 1). The molecular weight profiles of the F3 derivatives were afterward checked using a set of analytical gel permeation columns (Fig. 6B). F3 derivatives from HP-I and HB-L elute from the column as narrow and coincident peaks while HB-I presented a shoulder shifted towards the V_t_ (Fig. 6B), which reflects in a slightly lower *M*_*n*_value (Table 1). Nevertheless, the three F3 derivatives have showed remarkable molecular weight homogeneity, with F3 derivatives from HP-I and HB-I showing lower and HB-L higher molecular weight than their unfractionated parental heparins (Table 1).

The disaccharide composition of F3 derivatives were investigated via 1D ^1^H (Fig. 6C–E) and 2D ^1^H -^13^C HSQC (Supplementary Figure S3, Table 2) NMR spectra. All the three derivatives were structurally similar to their parental-unfractionated heparins (Fig. 6C–E), except for minor differences in the region of β-glucuronic acid + linkage region, especially in the spectrum of the derivative from HB-L (Supplementary Figure S3). Therefore, we successfully have produced preparations of the heparins with similar molecular weights differing exclusively in their disaccharide compositions in a way similar to their parental-unfractionated molecules.

### Anticoagulant activities of F3 derivatives

Once a reasonable amount of the F3 derivatives were gathered, we evaluated their *in vitro* anticoagulant activities via APTT, anti-FIIa and anti-FXa assays and then compared with their parental-unfractionated heparin batches (Fig. 6F–H). F3 derivative from HP-I and HB-I showed no significant differences (P > 0.05) in comparison with their parental-unfractionated batches (Fig. 6F–H). In contrast, the F3 derivative from HB-L presented a substantial increase in its anticoagulant effect, becoming significantly more potent (P < 0.05) than its parental-unfractionated batch on all the three assays (Fig. 6F–H).

Comparing the F3 derivatives among themselves we clearly observed those from HP-I and HB-L with similar anticoagulant effect (~170 IU mg^−1^), especially on their anti-FIIa and anti-FXa activities (Fig. 6F–H); however, the derivative from HB-I is noticeably less potent on all assays (<100 I.U. mg^−1^), possibly due to its structural characteristics such as the marked 6-desulfation of the α-glucosamine units. Through these results we can now establish a strict correlation between the disaccharide composition and anticoagulant activity of heparin excluding the influence of the molecular weight.

## Discussion

Currently heparin is defined by most pharmacopeias as a single biological drug, regardless the animal source used for its production^1^. Heparin was initially mass-produced from bovine lung and lately from porcine intestine^5^. Some Latin American and Islamic countries also employ heparin from bovine intestine as a therapeutic alternative^6^. However, the transition from HB-L to HP-I and the concomitant introduction of HB-I were not accompanied by new regulations or clinical evaluations by the health authorities.

Here we performed analytical assessments (361 batches) of pharmaceutical heparins obtained from these different animal sources (*viz.* porcine and bovine intestinal mucosa and bovine lung), which showed different physicochemical features such as their ionic properties and the lower molecular weight of HB-L. Besides, these heparins also presented differences in their disaccharide compositions; HP-I and HB-L present more alike structures, with similar sulfation patterns in their α-glucosamine units and 2-sulfated α-iduronic acid proportions; however, HB-L has considerably lower proportion of β-glucuronic acid. In contrast, HB-I shows a remarkably distinct structure, with higher 6-desulfated α-glucosamine and lower β-glucuronic acid linked to *N*-glucosamine *N*,3,6-trisulfated proportions. Despite of the structural resemblances between HP-I and HB-L, these heparins presented dissimilar anticoagulant activities, with HB-L potency close to HB-I and markedly lower than HP-I.

Differences among heparins obtained from different animal sources were recently reported elsewhere^21^,^22^,^23^.The results obtained via NMR spectroscopy presented in these articles also suggest that heparins from porcine and bovine intestine have dissimilar structures. However, none of these works thoroughly correlate the molecular weight and disaccharide composition of those different heparins with their anticoagulant and antithrombotic potencies, as reported here. Besides, these works assessed a limited number of heparin samples, differently of our work, which assessed several different pharmaceutical grade heparins commercially available for therapeutic use.

We also investigated the impact of the molecular weight on the anticoagulant activity of these different heparins. The fractions F3 derived from the three heparin types differ exclusively in their disaccharide units. F3 derivatives from HP-I and HB-L showed disaccharide compositions and anticoagulant activities more alike to each other. In contrast, HB-I, showed major differences in its structure, especially regarding the high proportion of 6-desulfated α-glucosamine residues, and remarkably lower (~50%) anticoagulant activity. These results clearly demonstrate the pivotal role of the molecular weight on the anticoagulant activity of heparin. A previous study showed it is possible to separate via ion-exchange chromatography HB-I polymers enriched in 6-sulfated α-glucosamine units, with improved anticoagulant activity^11^. Taking into consideration that heparin is a heterogeneous and polydisperse polymer, purification processes aiming for a specific molecular weight range or ionic property can result in preparations with improved anticoagulant potencies.

In the last decades the knowledge on the pharmaceutical and biochemical features of heparin increased notably, mainly as result of the advances in the analytical tools to investigate its complex structure (*i.e.* NMR) and biological effects^10^,^11^,^24^. These advances have allowed more precise and comprehensive quality control analysis of pharmaceutical preparations of heparin. An example of the necessity of these thorough quality assessments was the case of heparin contamination with oversulfated chondroitin sulfate, an event with great repercussion within the health community and associated with several deaths^25^. Another important advance has been the production of HP-I with enhanced specific activity (IU mg^−1^), which can be then administrated as lower doses (in weight basis)^5^ and hence requiring lower doses of protamine for its neutralization.

Clinical use of unfractionated heparin requires an extra caution because the dose necessary to produce the desired anticoagulant or antithrombotic effect is very close to those inducing adverse effects, especially bleeding. Furthermore, heparin therapy is usually employed in serious clinical situations, such as cardiovascular surgeries or prevention and treatment oh thromboembolism episodes. As demonstrated here and elsewhere^10^,^11^. HB-I requires about two-fold higher doses (in weight basis) than HP-I to achieve the same anticoagulant and antithrombotic effect. In consequence, HB-I will demand higher doses of protamine than HPI to be effectively neutralized, thus avoiding bleeding incidents. This was observed on *in vitro* assays^10^,^11^.

Few studies comparing the efficacy and/or safety of heparins obtained from different sources were already performed e.g.^26^,^27^. The most notable adverse effect reported was the increased incidence of thrombocytopenia associated with HB-L in comparison with HP-I^28^. The concomitant clinical use of HP-I and HB-I in Brazil precipitated several bleeding incidents during cardiovascular surgeries, drawing attention of the regulatory authorities^29^,^30^. The only clinical study comparing HP-I and HB-I was performed with patients undergoing hemodialysis^31^; in this study both heparins were similarly efficient and safe, but showed different anticoagulant activities due to an inadequate determination of the therapeutic formulation.

Our results thoroughly indicate that heparins from different animal sources consist in effective but distinct drugs, this is because: (1) HB-I presents distinct chemical structure and anticoagulant efficacy from HP-I; (2) HB-L and HP-I present preponderant structure more alike each other, however, their anticoagulant efficacy and physicochemical properties (mainly the molecular weight) significantly differ; (3) HB-L and HB-I have fairly similar anticoagulant effect, but greatly differ in their chemical structures.

More than one century has passed since the discovery of heparin, during this time, the understanding on its nature and uses has grown dramatically^32^. Even so, heparin stills continuously challenging us with new questions concerning its structure, mechanism of action, manufacturing, quality controls, etc. Nowadays, when similarity of biological drugs is a central theme in pharmacology, the heparins from different animal sources approached here show to us new challenges, perhaps as a paradigm to define thresholds separating distinct biological drugs.

## Methods

### Heparins and standard glycosminoglycans samples

Three hundred and sixty-one batches of pharmaceutical grade heparins were analyzed: 150 from porcine (HP-I) and 210 from bovine (HB-I) intestinal mucosa and one from bovine lung (HB-L). All HP-I and HB-I samples consisted of non-expired commercially available pharmaceutical preparations. Batches of HB-I were obtained from two and of HP-I from three different Brazilian suppliers. The HB-L sample was kindly given by Dr. Barbara Mulloy. All batches were analyzed by 1D ^1^H NMR spectrum. At least six batches of both HP-I and HB-I obtained from two suppliers (one for each type of heparin) were used for analysis of molecular weight distribution and determination of the anticoagulant activity. This assures correlation between the physicochemical properties and the biological activity. 6^th^ International Heparin Standard (2,145 units per vial, Lot No. 07/328) was obtained from the National Institute for Biological Standards and Control (NIBSC; Potters Bar, UK), dermatan sulfate was purchased from Sigma Aldrich (St. Louis, US) and oversulfated chondroitin sulfate (Lot No. HOM191) from United State Pharmacopeia (USP; Washington DC, USA).

### Anion-exchange chromatography

Heparins were analyzed via anion-exchange chromatography using an IonPac AS11-HC column (Dionex; Sunnyvale, USA) equilibrated with 10 mM Tris-HCl with 0.5 M NaCl, pH 7.4, linked to a HPLC system (Shimadzu; Kioto, Japan) as previously described^33^. Heparins (200 μg of each), standard heparin (200 μg), dermatan sulfate (50 μg) and oversulfated chondroitin sulfate (30 μg) were applied to the column, washed with equilibration buffer (10 mL) and then eluted using a linear gradient of 0.4 → 2.5 M NaCl (40 mL), at a flow rate of 0.5 mL.min^−1^. The eluent was continuously monitored via UV (A_215nm_). The background produced by the NaCl gradient was subtracted from the chromatograms obtained applying the glycosaminoglycans (GAGs).

### Determination of the molecular weight

Molecular weight distribution of heparins was determined via gel permeation chromatography using a set of TSK gel G4000 SW × 1 and G3000 SW × 1 columns, both 7.5 mm × 300 mm i.d. (Tosoh; Tokyo, Japan), linked to a HPLC system (Shimadzu) and equilibrated with 0.1 M ammonium acetate. The combination of G4000 and G3000 columns spread the molecular weight exclusion limits yielding well-marked separations. The heparins and standard GAGs (200 μg of each) were eluted with equilibration buffer at 40 °C, flow rate of 0.3 mL min^−1^ and monitored by differential refractive index. The columns were calibrated using a low-molecular-weight heparin molecular-weight standard (Lot No. 05/112) from NIBSC and a heparin sodium molecular-weight calibrant from USP (Lot No. FOL4830). Average molecular weight (*M*_*w*_), peak molecular weight (*Mp*), number average molecular weight (*M*_*n*_) and polydispersity degree (PD) were calculated as previously described^5^.

### Preparation of F3 derivatives

F3 derivatives of each heparin type were prepared via gel permeation chromatography using a Peptide HR 10//30 column (GE healthcare, Chicago, United States) linked to a HPLC system (Shimadzu, Tokyo, Japan) and equilibrated with 0.5 M ammonium acetate (pH 5.0). Heparins (20 mf of each type) were eluted with the equilibration buffer at a flow rate of 0.5 mL.min^−1^ and monitored by differential refractive index. Fractions of 0.5 mL were collected, pooled as indicated in Fig. 6A and lyophilized. Sodium salt of the F3 derivatives was obtained following Dowex 50 Wx8 (H^+^) exchange.

### Analysis of heparins by NMR

^1^H one-dimensional (1D) and ^1^H and ^13^C two-dimensional (2D) nuclear magnetic resonance (NMR) spectra of heparins batches were recorded using a Bruker DRX 800 MHz apparatus (Bruker; Billerica, USA) with a triple resonance probe, as detailed previously^13^,^15^,^16^. About 20 mg of each batch were dissolved in 0.5 mL 99.9% deuterium oxide (Cambridge Isotope Laboratories; Tewksbury, USA). All spectra were recorded at 35 °C with HOD suppression by presaturation. 1D ^1^H NMR spectrum of 361 batches were recorded with 64 scans and inter-scan delay of 1 second. 1D spectra of 20 batches of each HB-I and HP-I were weighted using AMIX software (Bruker) to generate concatenate fits. 2D phase sensitive ^1^H - ^1^H TOCSY (total correlated spectroscopy) and ^**1**^H - ^13^C HSQC (heteronuclear single quantum coherence) spectra were recorded using states-time proportion phase incrementation (TPPI) for quadrature detection in the indirect dimension. Phase-sensitive ^1^H-^1^H TOCSY spectra run as previously described^15^,^16^, with 4046 × 400 points with a spin-lock field of 10 kHz and a mix time of 80 ms.^1^H-^13^C HSQC spectra run with 1024 × 256 points and globally optimized alternating phase rectangular pulses (GARP) for decoupling^13^. Chemical shifts are displayed relative to external trimethylsilylpropionic acid at 0 ppm for ^1^H and relative to methanol for ^13^C.

### Activated partial thromboplastin time assay (APTT)

Human plasma (100 μL) and various concentrations of the heparins were incubated for 2 min at 37 °C with 100 μL of APTT reagent (kaolin bovine phospholipid reagent from Biolab-Merieux AS; Rio de Janeiro, Brazil). After incubation 100 μL of 25 mM CaCl_2_ was added to the mixture and the clotting time recorded in an Amelung KC4A coagulometer (Heinrich Amelung GmbH; Lemgo, Germany). The results where expressed as the ratio of clotting time in the presence (T) and absence (T_0_) of different volumes (2–10 μL) of the heparins samples (50 μg mL^−1^) or the 6^th^ International Heparin Standard (10 IU mL^−1^) and then fit to second order polynomial curves^31^. Anticoagulant potencies of the heparins samples as IU mg^−1^ (as dry weight) were derived from the parameters obtained in the 6^th^ International Heparin Standard fit.

### *In vitro* anti-FXa and anti-FIIa activities

The heparins were subject of amidolytic activity assessments of thrombin (FIIa) or factor Xa (FXa) by measuring the hydrolysis of chromogenic substrates as previously described^10^,^11^. Incubations were performed in 96-well plates with 10 nM antithrombin, 2.0 nM FXa or thrombin (all from Hematologic Technologies; Essex Junction, USA) and 0 to 2 μg mL^−1^ heparins (dry weight) in TS/PEG buffer (0.02 M Tris/HCl, 0.15 M NaCl and 1.0 mg mL^−1^ polyethylene glycol 8,000, pH 7.4). FXa or FIIa were added lastly to trigger the reaction. After incubation for 60 s at 37 °C, residual FXa or FIIa activities were determined by the addition of 100 μM of chromogenic substrates S-2765 or S-2238, respectively (both from Chromogenix; Molndal, Sweden). Absorbance (405 nm) was recorded for 300 s in a Thermomax Microplate Reader (American Devices; Sunnyvile, California). Anti-FXa and anti-FIIa activities were obtained by parallel line assays against the 6^th^ International Heparin Standard (NIBSC) using SoftMax Pro5.4.1 software (American Devices; Sunnyvile, California) and validated methods recommended by the pharmacopeias. When the results obtained with HB-I were adjusted to IU mL^−1^ in the assays, they generate coincident curves with the 6^th^ International Heparin Standard (NIBSC).

### Assessment of venous antithrombotic activity

Antithrombotic activity was measured in Wistar rats using rabbit brain thromboplastin (Biolab-Merieux AS, Rio de Janeiro, Brazil) as thrombogenic stimulus^18^. Animal experiments were carried out in accordance with the institutional guidelines and regulations. All experimental protocols were approved by the licensing committee of the Federal University of Rio de Janeiro (Protocol No. UFRJ-CCS-015). Rats (both sexes, ~200 g body weight, five animals per dose) were anesthetized with an intramuscular injection of 100 mg kg^−1^ body weight of ketamine (Cristalia; São Paulo, Brazil) and 16 mg kg^−1^ body weight of xylazine (Bayer AS; Leverkusen, Germany). Different doses of the heparins were infused into the right carotid artery and were letting to circulate for 5 min. The inferior vena cava was isolated and then thromboplastin (5 mg kg^−1^ body weight) was slowly injected intravenously and after 1 min 0.7 cm of isolated vena cava was clamped off using distal and proximal sutures. After 20 min stasis the thrombus formed inside the occluded segment was carefully pulled out, washed with phosphate-buffered saline, dried for 1 h at 60 °C and weighted. The results were expressed as a ratio of thrombus weight in the presence and absence of the heparins.

### Bleeding effect

Wistar rats (both sexes, ~200 g body weight) were anesthetized as described above. A cannula was inserted into the right carotid artery for administration of the heparins at a dose of ~200 IU kg^−1^ body weight. After heparins circulate for 5 min the tail tip was cut (3 mm of diameter) and carefully immersed in 30 mL distilled water at room temperature. Blood loss was quantified 60 min later by measuring the hemoglobin dissolved in the water via spectrophotometry (absorbance 540 nm) and then deduced from a standard curve based on known volumes of blood. The results were expressed as ratios of blood loss of heparin treated and saline treated animals^19^. In some assays the heparins were neutralized after circulate for 5 minutes with 2 mg kg^−1^ body weight of protamine sulfate (Valeant, São Paulo, Brazil) and then the bleeding assay was carried out as described above. One mg of protamine neutralizes 100 IU of heparin^34^.

### Statistical analysis

Statistical analyses were performed using Sigma Stat software (Systat; San Diego, USA) and OriginPro 8 software (OriginLab; Northtampton, USA).

## Additional Information

**How to cite this article**: Tovar, A. M. F. *et al*. Structural and haemostatic features of pharmaceutical heparins from different animal sources: challenges to define thresholds separating distinct drugs. *Sci. Rep.* **6**, 35619; doi: 10.1038/srep35619 (2016).

## Supplementary Material

Supplementary Information

## Figures and Tables

**Table 1:** Average molecular weight (*M*_*w*_), peak molecular weight (*M*_*p*_), number average molecular weight (*M*_*n*_) and polydispersity degree (PD) of HP-I. HB-I and HB-L (mean ± SD).

	means ± SD		
	HP-I	HB-I	HB-L
**A) Unfractionated**	(n = 6)	(n = 6)	(n = 1)
*M*_*w*_	17,721 ± 146^a^	17,717 ± 704^a^	12,729
*M*_*p*_	16,900 ± 32^b^	15,892 ± 526^b^	10,500
*M*_*n*_	13,841 ± 230^b^	14,946 ± 559^b^	9,737
PD	1.24^b^	1.19^b^	1.31
**B) F3 derivatives**			
*M*_*w*_	15,142	14,558	14,924
*M*_*p*_	13,800	13,900	13,600
*M*_*n*_	13,777	12,821	13,773
PD	1.10	1.14	1.08

^a^No significant different for HP-I *vs* HB-I.

^b^Significant different (P < 0.05).

**Table 2:** Proportions (mean or mean ± SD) of the constitutive units of HP-I, HB-I and HB-L determined via solution NMR.

Signal number^a^	Structure^e^	Unfractionated			F3 derivatives		
		HP-I (n = 3)	HB-I (n = 3)	HB-L (n = 1)	HP-I (n = 1)	HB-I (n = 1)	HB-L (n = 1)
**A) α-glucosamine units**^b^							
1	**α-Glc*****N*****,3,6-triS**-[?]	4.58 ± 0.69	2.29 ± 1.19	3.41	5.16	2.56	3.90
2-a	**α-Glc*****N*****,6-diS**-[α-IdA-2S]	68.50 ± 4.84^f^	48.27 ± 1.35^f^	81.91	70.70	46.29	78.66
2-b^c^	**α-Glc*****N*****Ac**-[β-GlcA]	6.94 ± 2.42	3.74 ± 1.22	2.15	6.86	3.50	3.12
3	**α-Glc*****N*****S**-[α-IdA]	8.07 ± 2.47	4.52 ± 2.20	4.21	6.87	6.48	3.31
4	**α-Glc*****N*****S**-[β-GlcA]	9.08 ± 0.84^f^	13.86 ± 0.58^f^	7.21	8.75	15.39	9.19
5	**α-Glc*****N*****S**-[α-IdA-2S]	2.81 ± 1.01^f^	27.51 ± 2.40^f^	1.11	1.56	25.78	1.82
**B) α-iduronic acid units**^b^							
6	**α-IdA2S**	77.49 ± 2.47	75.28 ± 3.02	90.14	73.98	75.20	85.46
7	**α-IdA**-[α-Glc*N*,6S]	4.50 ± 1.79	1.36 ± 0.07	2.56	6.19	1.94	2.37
8	**α-IdA**	1.44 ± 0.47	4.18 ± 1.57	0.53	1.65	2.03	0.85
	**∑ α-IdA**	83.43	80.82	93.23	81.82	79.17	88.68
**C) β-glucuronic acid**^b^							
9	**β-GlcA**-[α-Glc*N*,3,6-triS]	2.42 ± 0.32^f^	0.48 ± 0.12^f^	0.69	3.04	0.18	0.28
10	**β-GlcA**-[α-Glc*N*Ac]	5.09 ± 1.53^f^	10.17 ± 1.14^f^	2.44	7.37	11.26	5.60
11	**β-GlcA**-[α-Glc*N*S]	8.02 ± 0.49^f^	6.62 ± 0.56^f^	3.34	7.42	7.96	5.43
12	**β-GlcA2S**-[?]	0.55 ± 0.23^f^	1.91 ± 0.21^f^	0.30	0.35	1.43	<0.01
	**∑ β-GlcA**	16.08	19.18	6.77	18.18	20.83	11.31
**D) Linkage region**^d^							
13	**β-Gal**	1.02 ± 0.31	0.60 ± 0.06	0.28	1.36	0.63	0.75
14	**β-Xyl**	0.87 ± 0.44	0.41 ± 0.33	0.16	0.81	0.69	0.18
15	**β-GlcA**-[β-Gal]	2.46 ± 1.57	1.96 ± 1.21	0.59	3.51	1.55	1.04

^a^See Panels B–D, Fig. 3.

^b^Results are express as percentage of the total α-glucosamine or hexuronic acid units (α-iduronic + β-glucuronic acids).

^c^The proportion of the α-Glc*N*Ac residue were calculated by integration of H2/C2 signals at 3.86/56.2 ppm and subtracted from the anomeric signal representing both α-Glc*N*Ac and α-Glc*N*S.

^d^Results express as percentage of the total units.

^e^The reported structure is in bold and the subsequent unit is in bracket.

^f^Values significantly different for porcine *vs* bovine intestinal heparins (P < 0.05).
