# Supplementary material for: Structural and haemostatic features of pharmaceutical heparins from different animal sources: challenges to define thresholds separating distinct drugs
Source: Sci Rep. 2016 Oct 18;6:35619. doi: 10.1038/srep35619 (PMC5067489; doi:10.1038/srep35619)
Supplement: Supplementary Information [file srep35619-s1.pdf]

Structural and haemostatic features of pharmaceutical heparins from different animal sources: challenges to define thresholds separating distinct drugs

Ana M. F. Tovar, Gustavo R.C. Santos, Nina V. Capillé, Adriana A. Piquet, Bianca F. Glauser, Mariana S. Pereira, Eduardo Vilanova, Paulo A.S. Mourão

Supplementary Information

Table S1. Capacity of retention (mean ± SD) of HP-I, HB-I and HB-L on the anion exchange chromatography.

Table S2. Average molecular weight (M_w), proportions of molecular weight (M) lower than 24,000 Da and molecular weight ratios 8,000 $\rightarrow$ 16,000 Da and 16,000 $\rightarrow$ 24,000 Da measured for HP-I and HB-I.

Table S3. ^{1}H and ^{13}C chemical shifts of the anomeric protons and carbons of the constitutive units of HP-I, HB-I and HB-L.

Table S4. Anticoagulant activity (mean ± SD) of HP-I, HB-I and HB-L batches, parental batches of F3 derivatives and F3 derivatives.

Figure S1. 1D ^{1}H NMR spectra of mixtures containing increasing amounts of HB-I added to a fixed amount of HP-I.

Figure S2. Phase-sensitive ^{1}H - ^{1}H TOCSY spectra of HB-L and HB-I.

Figure S3. 2D ^{1}H - ^{13}C HSQC NMR spectra of F3 derivatives from HB-I, HP-I and HB-L and their parental batches.

NaCl - M	% of total heparin sample eluted		
	**HP-I (n = 6)**	**HB-I (n = 5)**	**HB-L (n = 1)**
**≤ 1.85**	10.18 ± 0.44[a]	33.39 ± 2.81[a]	18.90
**≤ 1.90**	27.71 ± 0.56[a]	56.20 ± 2.54[a]	35.47
**≤ 1.95**	69.89 ± 0.27[a]	86.66 ± 1.28[a]	71.60
**≤ 2.00**	94.98 ± 0.11	98.38 ± 0.31	94.25
**NaCl concentration (as M) at the peak elution**	1.928 ± 0.004[a]	1.865 ± 0.010[a]	1.921

[a]Significant different (P < 0.05) for HP-I *vs* HB-I.

Table S1. Capacity of retention (mean ± SD) of HP-I, HB-I and HB-L on the anion exchange chromatography.

		M_w (Da)	*M* > 24,000 Da (% of total)	Ratio *M* 8,000 → 16,000 Da / *M* 16,000 → 24,000 Da
**Recommendation of the USP[b]**		15,000 - 19,000	<20	> 1.0
**HP-I**	Batch 1	17,000	13.96	1.11
	Batch 2	17,114	14.29	1.10
	Batch 3	17,025	14,00	1.10
	Batch 4	17,399	14,36	1.05
	Batch 5	17,142	14.36	1.09
	Batch 6	17,045	14.04	1.10
**HB-I**	Batch 1	18,791	20.07	0.95
	Batch 2	17,275	14.88	1.32
	Batch 3	17,167	14.56	1.32
	Batch 4	17,964	16.78	1.09
	Batch 5	16,958	14.28	1.37
	Batch 6	18,148	17.54	1.10

[a]Colums were calibrated using low-molecular-weight heparin and molecular-weight standard heparin calibrants, for further detail see the legend of Fig.2. The results may be slightly different using only the unfractionated heparin calibrant.
[b]See [19].

Table S2. Average molecular weight (M_w), proportions of molecular weight (*M*) lower than 24,000 Da and molecular weight ratios 8,000 → 16,000 Da and 16,000 → 24,000 Da measured for HP-I and HB-I[a].

Signal number[a]	Structure[c]	$^1H/^{13}C$-chemical shifts (ppm)		
		HP-I	HB-I	HB-L
**A) α-glucosamine units**				
1	**α-Glc*N*,3,6-triS-**[?]	5.52/98.1	5.55/98.2	5.51/98.3
2[b]	**α-Glc*N*,6-diS-**[α-IdA-2S] + **α-Glc*N*Ac-**[β-GlcA]	5.40/98.6	5.42/98.6	5.40/98.6
3	**α-Glc*N*S-**[α-IdA]	5.36/97.4	5.39/97.5	5.34/97.5
4	**α-Glc*N*S-**[β-GlcA]	5.58/99.5	5.58/99.5	5.57/99.6
5	**α-Glc*N*S-**[α-IdA-2S]	5.31/98.9	5.31/99.3	5.30/99.2
**B) α-iduronic acid**				
6	**α-IdA2S**	5.22/101.0	5.24/101.2	5.22/101.2
7	**α-IdA-**[α-Glc*N*,6S]	5.01/103.9	5.03/104.0	5.01/104.0
8	**α-IdA**	4.95/103.7	4.96/103.7	4.95/103.8
**C) β-glucuronic acid**				
9	**β-GlcA-**[α-Glc*N*,3,6-triS]	4.63/102.9	-	4.62/103.1
10	**β-GlcA-**[α-Glc*N*Ac]	4.52/104.2	4.54/104.2	4.52/104.2
11	**β-GlcA-**[α-Glc*N*S]	4.61/103.8	4.62/103.9	4.61/103.8
12	**β-GlcA2S-**[?]	-	4.72/102.7	4.70/102.7
**D) Linkage region**				
13	**β-Gal**	5.54/103.3	4.55/103.4	4.54/103.4
14	**β-Xyl**	4.46/104.8	4.48/104.9	4.44/105.0
15	**β-GlcA-**[β-Gal]	4.68/105.7	4.69/105.8	4.66/105.7

[a]See panels B - D, Fig. 3.

[b]These two units have similar ^1H and ^{13}-chemical shifts of their anomeric signals.

[c]The reported structure is in bold and the subsequent unit is in bracket.

Table S3. ^1H and ^{13}C chemical shifts of the anomeric protons and carbons of the constitutive units of HP-I, HB-I and HB-L.

Heparin	APTT[a]	anti-FIIa[a]	anti-FXa[a]	anti-FIIa/ anti-FXa[e]
**HP-I (batches)[b]**	203.0 ± 5.3 / n = 6	185.5 ± 14.1 / n = 8	195.2 ± 18.3 / n = 11	0.95
**HB-I (batches)[b]**	88.7 ± 8.2 / n = 7	90.8 ± 10.2 / n = 9	91.7 ± 15.0 / n = 11	0.99
**HB-L (batches)[c]**	97.2 ± 8.6 / n = 9[d]	133.8 ± 15.5 / n = 8	121.9 ± 16.4 / n = 10	1.09
**HP-I (parental)[c]**	181.8 ± 7.8 / n = 4	165.8 ± 12.6 / n = 4	173.3 ± 10.8 / n = 6	0.95
**HB-I (parental)[c]**	105.5 ± 11.5 / n = 3	121.7 ± 10.9 / n = 4	105.3 ± 10.1 / n = 6	1.15
**HB-L (parental)[c]**	97.2 ± 8.6 / n = 9[d]	133.8 ± 15.5 / n = 8	121.9 ± 16.4 / n = 10	1.09
**F3 HP-I[c]**	168.1 ± 6.8 / n = 3	167.4 ± 9.9 / n = 4	164.5 ± 4.3 / n = 3	1.01
**F3 HB-I[c]**	89.4 ± 6.7 / n=3	105.2 ± 15.7 / n = 4	83.5 ± 10.6 / n = 4	1.26
**F3 HB-L[c]**	130.7 ± 4.7 / n=4[d]	166.4 ± 9.4 / n = 5	168.7 ± 9.5 / n = 3	0.98

[a] Anticoagulant assays expressed in IU mg^{-1} (mean ± SD / number of assays).

[b] n represents the number of different batches analyzed.

[c] n represents the number of replicates performed with the same batch.

[d] Values significantly lower ($P < 0.05$) than those obtained in the other assays.

[e] Ratio.

Table S4. Anticoagulant activity (mean ± SD) of HP-I, HB-I and HB-L batches (batches), parental batches of F3 derivatives (parental) and F3 derivatives (F3).

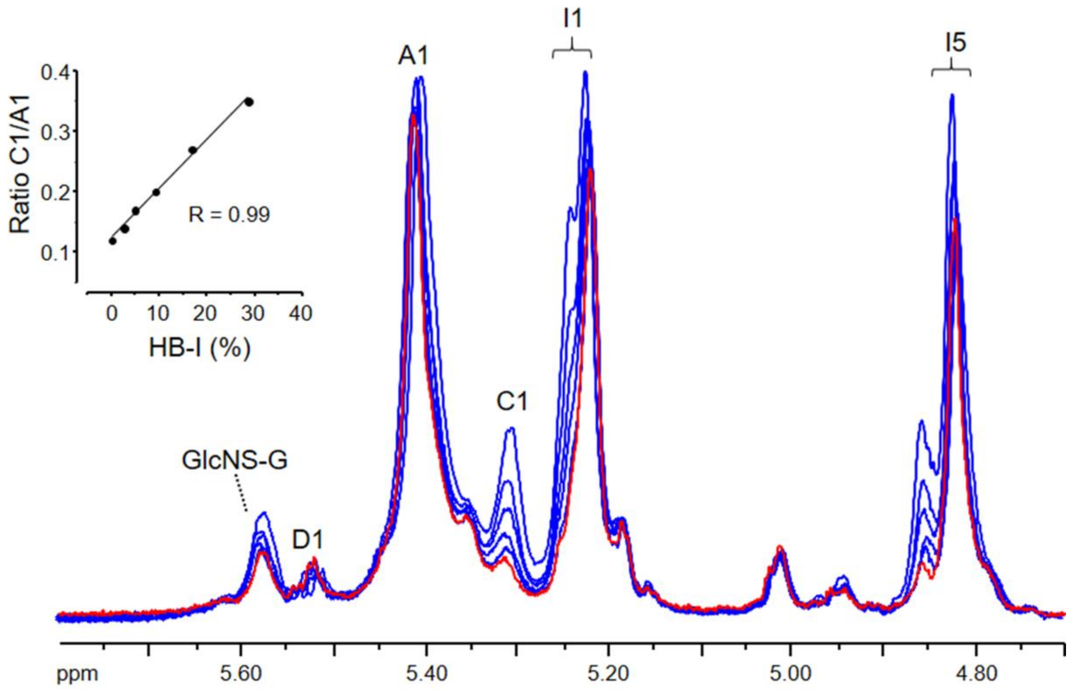

Figure S1. 1D ^{1}H NMR spectra (5.75 - 4.70 ppm region) of mixtures containing increasing amounts of HB-I added to a fixed amount of HP-I. Increasing quantities of HB-I (0.5 $\rightarrow$ 8.0 mg, in blue) were successively added to 20 mg of HP-I (in red) dissolved in 0.5 mL of 99.9% D_2O. See the legend of Fig. S1 for the nomenclature of the signals. The inset shows the ratio of peak integral of C1/A1 at different concentrations of HB-I (%) added to HP-I. This assay enables to detect mixtures of HB-I and HP-I up to 4%.

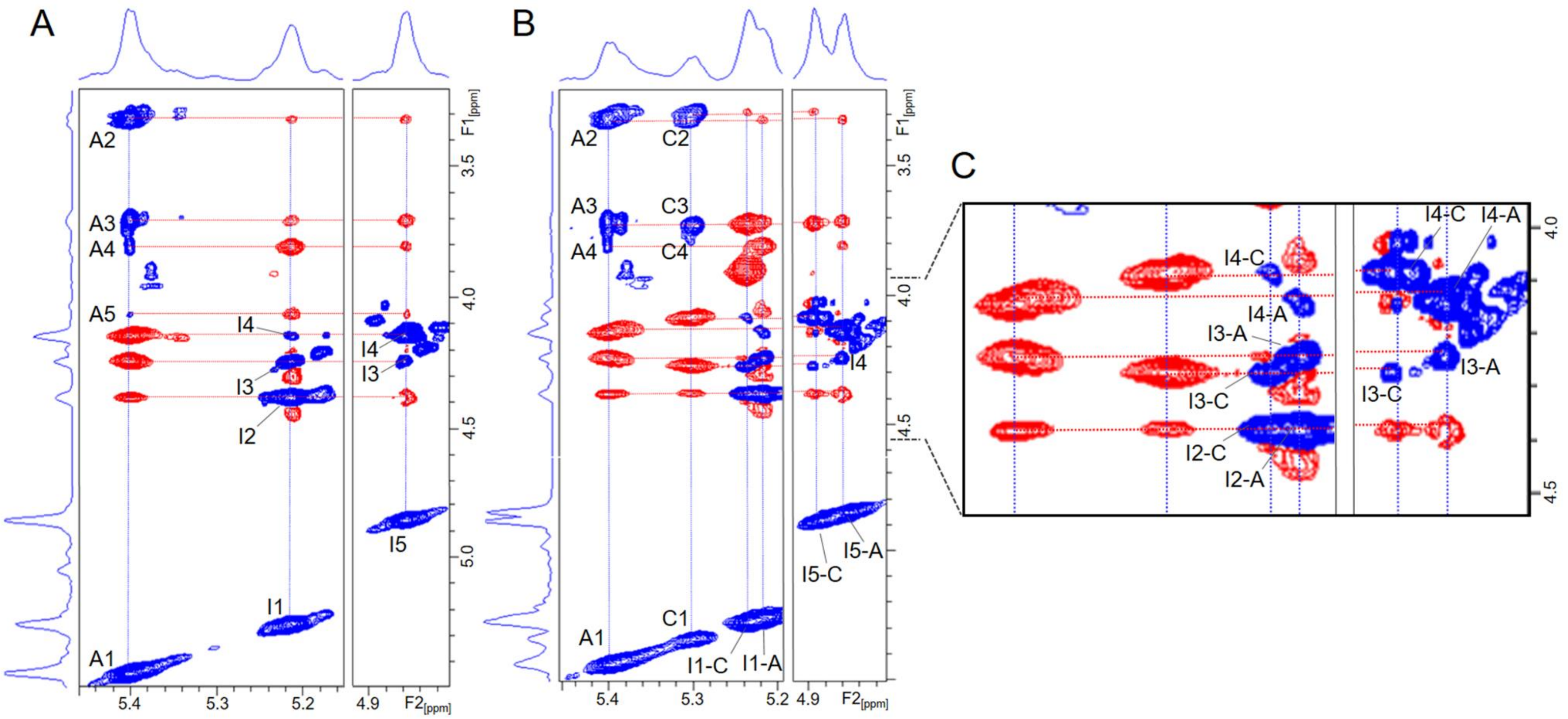

Figure S2. Phase-sensitive ^{1}H - ^{1}H TOCSY spectra of HB-L (A) and HB-I (B and C). These spectra discern signals of intra-residue spin systems from those of inter-residue ROEs. Saccharide connections were determined correlating the anti-phase (indicated in red and their connections by the horizontal dotted lines) and in-phase (shown in blue and their connections indicated by the vertical dotted lines) signals. The most intense anti-phase signal shows the linkage in the subsequent sugar residue. In the HB-L spectrum (A) *N*,6-disulfated α-glucosamine units (spin system A) connect with 2-sulfated α-iduronic acid residues (spin system I). A higher level of complexity can be observed in the spectrum of HB-I (B): residues of 6-desulfated α-glucosamine (spin system C) connect with the slightly downfield-shifted residues of 2-sulfated α-iduronic acid (namely I-C) while glucosamine *N*,6-disulfated (unit A) connects with the slightly upfield-shifted residues (namely I-A). These connections are amplified in the expansion shown in panel C. ^{1}H - ^{1}H NOESY spectra showed similar results but inter-residues signals are in-phase and thus not easily distinguishable from intra-residues signals (data not shown). Phase-sensitive ^{1}H - ^{1}H TOCSY spectra unambiguously demonstrate the origin of double signals in the spin system of 2-sulfated α-iduronic acid observed in the ^{1}H NMR spectrum of HB-I.

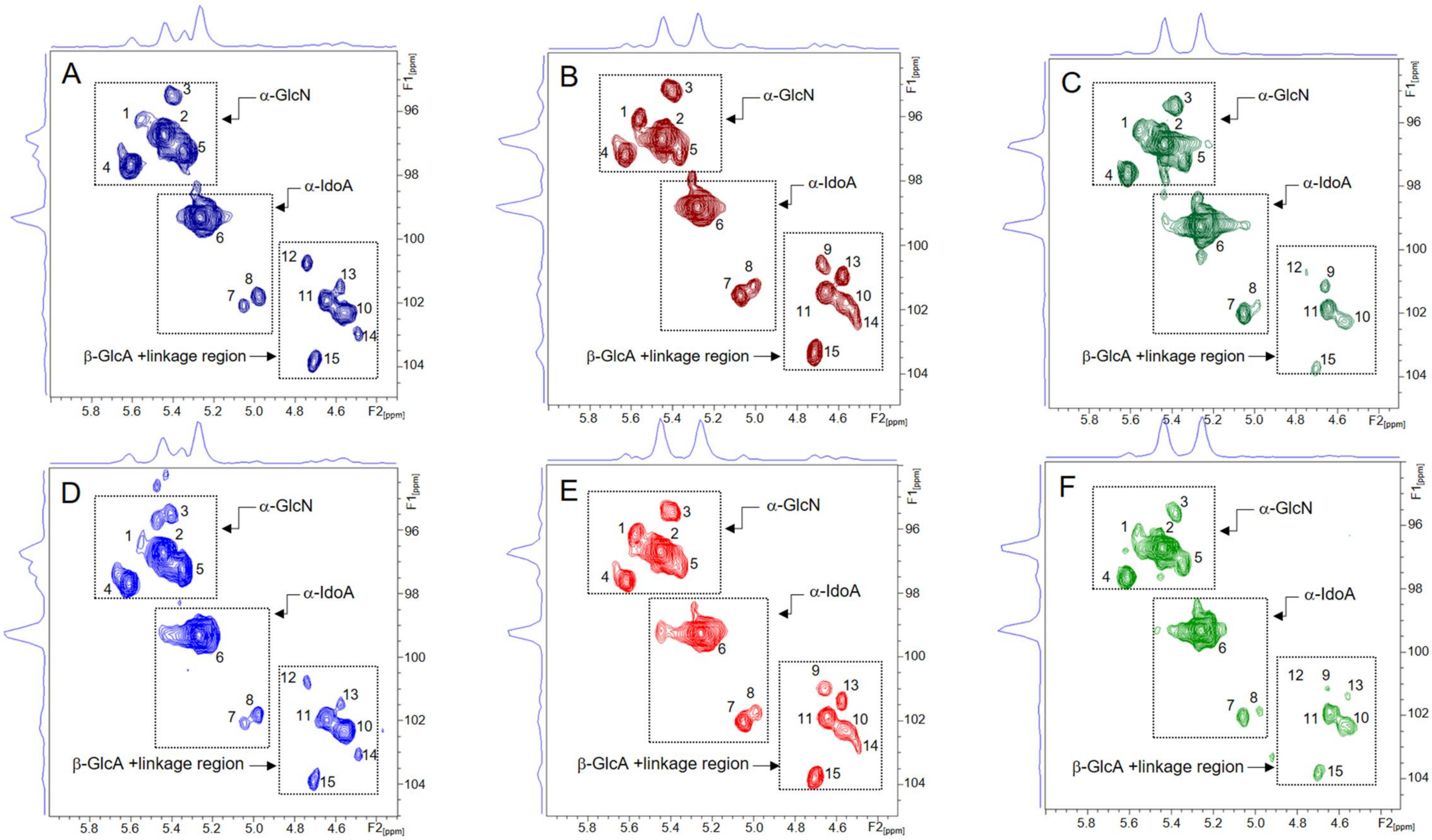

Figure S3. 2D ^{1}H - ^{13}C HSQC NMR spectra (strips of the anomeric region) of F3 derivatives from HB-I (A, in dark blue), HP-I (B, in dark red) and HB-L (C, in dark green) and their parental HB-I (D, in light blue), HP-I (E, in light red) and HB-L (F, in light green). Note the similarities among the spectra of F3 derivatives and their respective parental batches. See Table S3 for the chemical shifts and the legend for Fig. 3 for nomenclature of the signals and further details.
